# Datasets of YY1 expression in rheumatoid arthritis patients

**DOI:** 10.1016/j.dib.2016.11.046

**Published:** 2016-11-18

**Authors:** Jinpiao Lin, Yujue He, Junmin Chen, Zhiyong Zeng, Bin Yang, Qishui Ou

**Affiliations:** a Department of Laboratory Medicine, The First Affiliated Hospital of Fujian Medical University, China; b The Genetic Diagnostic Laboratory, The First Affiliated Hospital of Fujian Medical University, China; c Department of Hematology and Rheumatology, The First Affiliated Hospital of Fujian Medical University, China

**Keywords:** Rheumatoid arthritis, Interleukin-6, CRP, ESR

## Abstract

The data presented in this article are related to the research article entitled “A critical role of transcription factor YY1 in rheumatoid arthritis by regulation of interleukin-6” (J. Lin, Y. He, J. Chen, Z. Zeng, B. Yang, Q. Ou, 2016) [1]. The article describes YY1 overexpression is specific for RA, but not for SLE, SS, DM or MCTD. In early RA, YY1 expression is also increased. In asymptomatic subjects with RF or ACPA positive who have high risk for developing RA, the YY1 expression is not increased obviously. Moreover, YY1 expression is positively correlated with serum CRP or ESR. In RA patients treated with anti-IL-6R monoclonal Ab tocilizumab, there is no significant difference in YY1 expression after IL-6 blocking therapy.

**Specifications Table**

**Table:** 

Subject area	Biology
More specific subject area	Arthritis and Rheumatology
Type of data	Figures
How data was acquired	Real-time Polymerase Chain Reaction (Applied Biosystems PCR System 7500); Correlation analysis
Data format	Analyzed
Experimental factors	Peripheral blood mononuclear cells (PBMC) from RA, SLE, SS, DM or MCTD patients were freshly isolated. PBMC from early RA and established RA patients; PBMC from RA patients treated with tocilizumab were also collected. RNA was extracted from PBMC.
Experimental features	YY1 expression in autoimmune diseases or in RA patients treated with tocilizumab was detected by real-time PCR.
Data source location	The First Affiliated Hospital of Fujian Medical University, Fuzhou, China
Data accessibility	Data are presented in this article

**Value of the data**

• These data can be compared to other scientific data addressing the role of YY1 in other disease.
• These data provide insight into the function of YY1 in IL-6 production which can be of value for research groups from related fields.
• These data allow other researchers to extend the role of YY1 in RA study.

## 1 Data

**Fig. 1:**
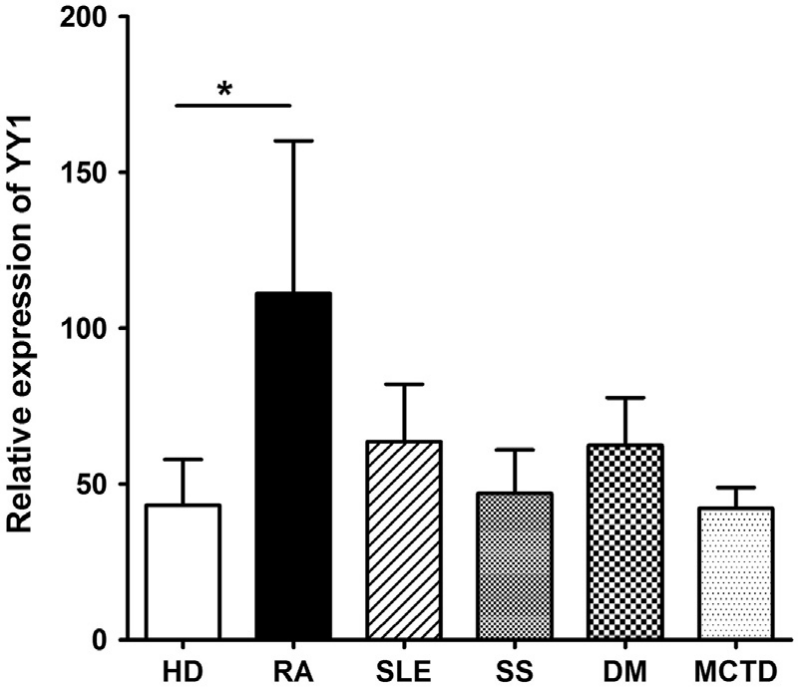
YY1 expression in PBMC from RA, SLE, SS, DM and MCTD patients. YY1 expression was over-expressed in RA compared to SLE, SS, DM and MCTD patients. HD: healthy donors; RA: rheumatoid arthritis; SLE: systemic lupus erythematosus; SS: Sjogren׳s syndrome; DM: dermatomyositis; MCTD: mixed connective tissue disease. ^⁎^*P*<0.05.

**Fig. 2:**
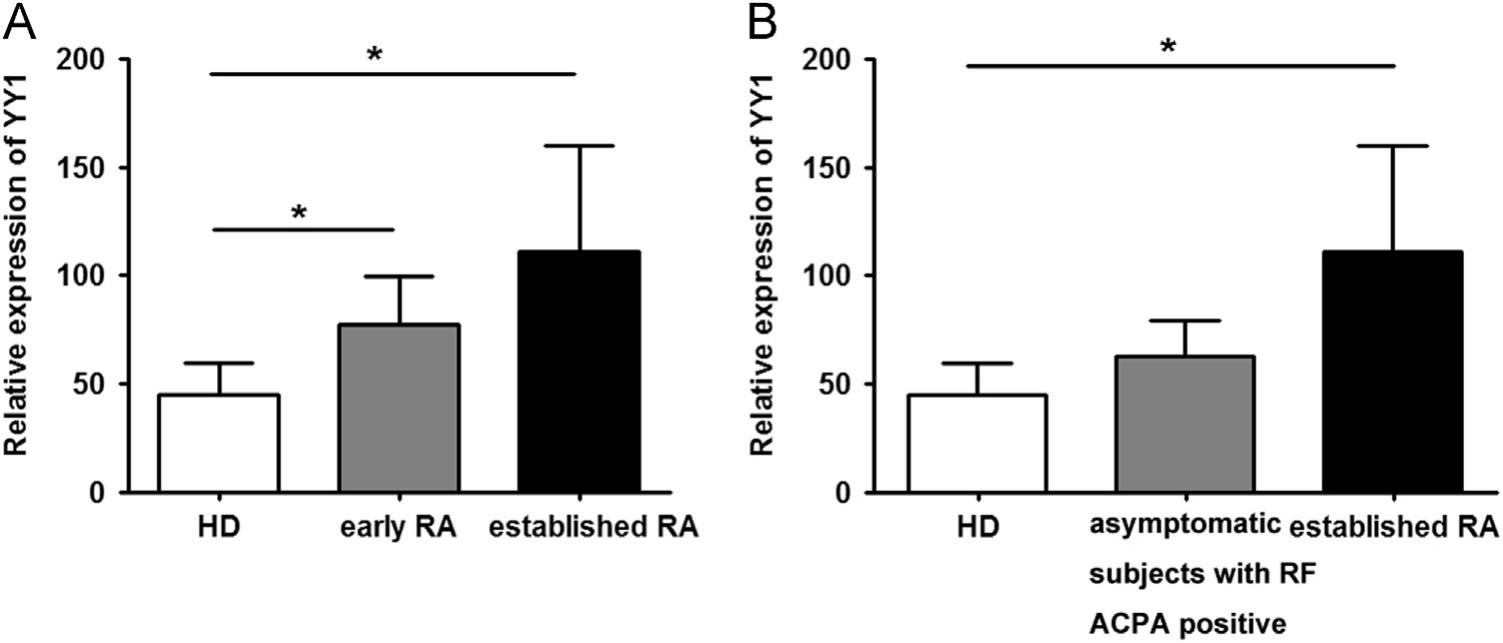
Increased expression of YY1 in early RA, but not in asymptomatic subjects with RF or ACPA positive. **A,** YY1 expression in early RA and established RA. **B,** YY1 expression in asymptomatic subjects with RF or ACPA positive. HD: healthy donors. RF: rheumatoid factor; ACPA: anti-citrullinated protein antibodies. ^⁎^*P*<0.05.

**Fig. 3:**
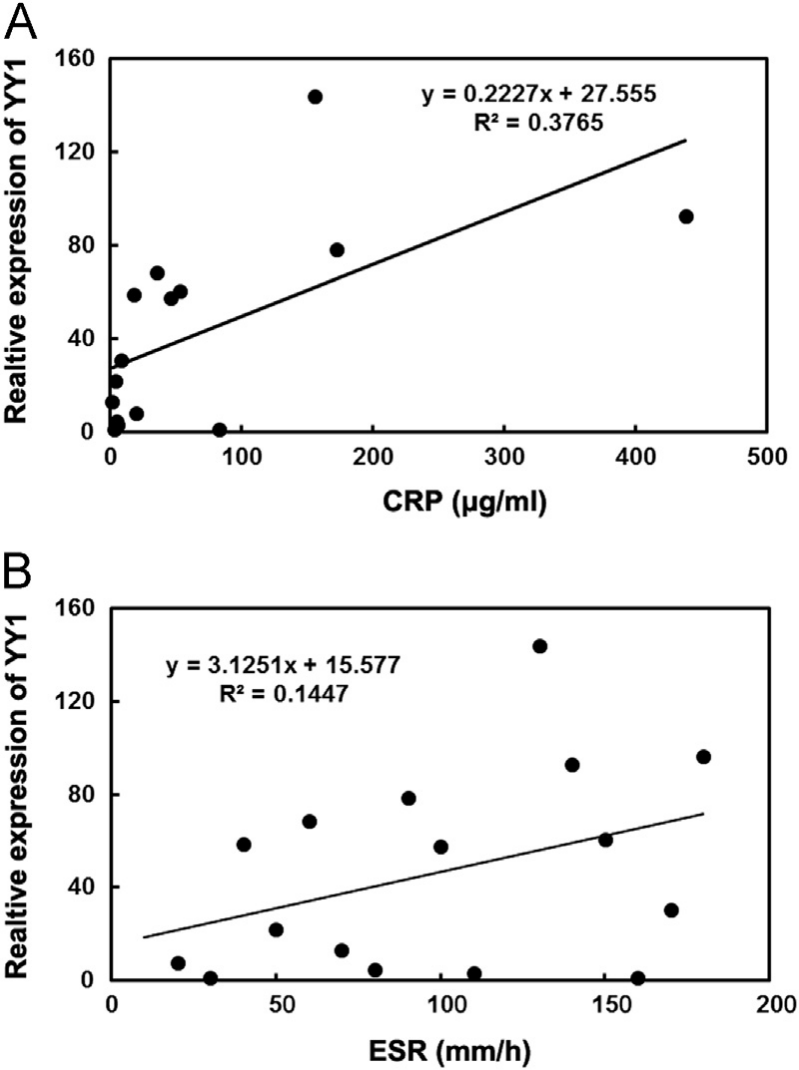
Positive correlation between YY1 expression and CRP or ESR. **A,** Correlation analysis of YY1 expression and CRP. **B**, Correlation analysis of YY1 expression and ESR. CRP: C-reaction protein; ESR: erythrocyte sedimentation rate.

**Fig. 4:**
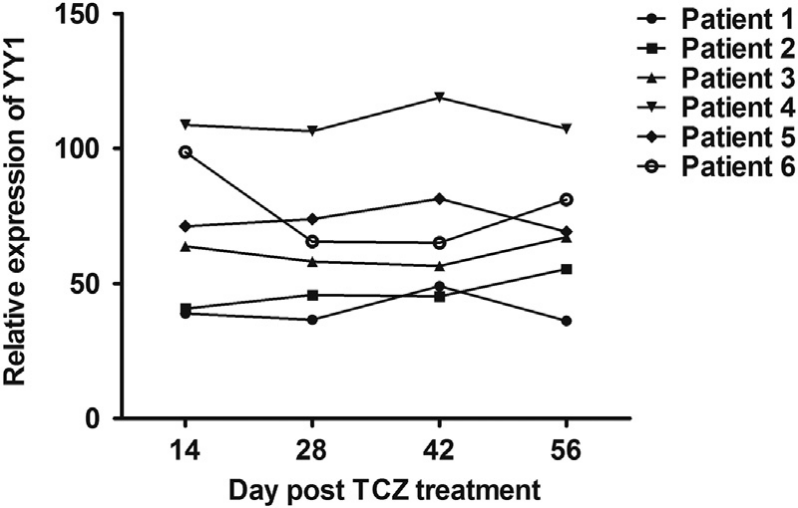
YY1 expression in RA patients treated with tocilizumab. PBMC from RA patients 14, 28, 42 and 56 days’ post tocilizumab treated was collected for detection of YY1 expression.

Data describes YY1 expression in RA, SLE, SS, DM and MCTD patients. The data show YY1 is overexpressed in RA, but not in SLE, SS, DM or MCTD patients (Fig. 1). YY1 expression is also increased in early RA compared to healthy control (Fig. 2A), however, YY1 expression in asymptomatic subjects with RF or ACPA positive is not increased significantly (Fig. 2B). Furthermore, YY1 expression is positively correlated with serum CRP or ESR in RA (Fig. 3). However, in RA patients treated with anti-IL-6R monoclonal Ab tocilizumab, the YY1 expression after IL-6 blocking therapy is not increased significantly (Fig. 4).

## 2 Experimental design, materials and methods

### 2.1 Patients

20 patients with systemic lupus erythematosus (SLE) fulfilled the 1997 revised American College of Rheumatology criteria [2], 10 patients with Sjogren׳s syndrome (SS) fulfilled the American–European Group Criteria [3], 10 patients with dermatomyositis (DM) fulfilled criteria described by Medsger [4], 8 patients with mixed connective tissue disease (MCTD) fulfilled the classification criteria described by Alarcon-Segovia [5] were enrolled for the study. Peripheral blood mononuclear cells (PBMC) from the patients was isolated by Ficoll. PBMC from early RA patients and asymptomatic subjects with RF or ACPA positive, PBMC from 6 RA patients before and after treated with tocilizumab (TCZ) for two months were also collected. All the PBMC collected was for detection of YY1 expression. Study protocols were approved by the Institutional Medical Ethics Review Board of the First Affiliated Hospital of Fujian Medical University, Fuzhou, China.

### 2.2 Real-time PCR analysis

Total RNA was extracted from PBMC cells, and real-time PCR was performed as previously described by us [6], [7]. The primers used in this study was as described by us [1].

### 2.3 Statistical analysis

Differences between groups was analyzed by unpaired Student׳s *t*-test. Correlation analyses was done by Microsoft Excel. For all statistical analyses, *P* values less than 0.05 was considered statistically significant. All statistical analyses were performed using GraphPad Prism 5.0 (GraphPad Software, San Diego, CA).

## References

[1] Lin J., He Y., Chen J. (2016). A critical role of transcription factor YY1 in rheumatoid arthritis by regulation of interleukin-6. J. Autoimmun..

[2] Hochberg M.C. (1997). Updating the American College of Rheumatology revised criteria for the classification of systemic lupus erythematosus. Arthritis Rheum..

[3] Vitali C., Bombardieri S., Jonsson R. (2002). Classification criteria for Sjogren׳s syndrome: a revised version of the European criteria proposed by the American-European Consensus Group. Ann. Rheum. Dis..

[4] Medsger T.A., Oddis C.V. (1995). Classification and diagnostic criteria for polymyositis and dermatomyositis. J. Rheumatol..

[5] Alarcon-Segovia D. (1982). Mixed connective tissue disease: some statements. Clin. Rheumatol..

[6] Lin J., Huo R., Xiao L. (2014). A novel p53/microRNA-22/Cyr61 axis in synovial cells regulates inflammation in rheumatoid arthritis. Arthritis Rheumatol..

[7] Lin J., Zhou Z., Huo R. (2012). Cyr61 induces IL-6 production by fibroblast-like synoviocytes promoting Th17 differentiation in rheumatoid arthritis. J. Immunol..

